# Predicting COVID-19 Sepsis Outcomes: Roles of IL-6, Cardiac Biomarkers, Clinical Factors, and Vaccination Status and Exploratory Analysis of Tocilizumab Therapy in an Eastern European Cohort

**DOI:** 10.3390/v17091168

**Published:** 2025-08-27

**Authors:** Diana-Maria Mateescu, Adrian-Cosmin Ilie, Ioana Cotet, Camelia-Oana Muresan, Ana-Maria Pah, Marius Badalica-Petrescu, Stela Iurciuc, Maria-Laura Craciun, Adrian Cote, Alexandra Enache

**Affiliations:** 1 Doctoral School, Department of General Medicine, “Victor Babes” University of Medicine and Pharmacy, Eftimie Murgu Square 2, 300041 Timişoara, Romania; diana.mateescu@umft.ro (D.-M.M.); ioana.cotet@umft.ro (I.C.); 2 Department of Public Health and Sanitary Management, “Victor Babeş” University of Medicine and Pharmacy, Eftimie Murgu Square 2, 300041 Timişoara, Romania; ilie.adrian@umft.ro; 3 Legal Medicine, Timișoara Institute of Legal Medicine, 300610 Timișoara, Romania; muresan.camelia@umft.ro (C.-O.M.); enache.alexandra@umft.ro (A.E.); 4 Ethics and Human Identification Research Center, “Victor Babeş” University of Medicine and Pharmacy, Eftimie Murgu Square 2, 300041 Timișoara, Romania; 5 Discipline of Forensic Medicine, Bioethics, Deontology, and Medical Law, Department of Neuroscience, “Victor Babeş” University of Medicine and Pharmacy, 300041 Timișoara, Romania; 6 Cardiology Department, “Victor Babes” University of Medicine and Pharmacy, Eftimie Murgu Square 2, 300041 Timişoara, Romania; anamaria.pah@umft.ro (A.-M.P.); marius.badalica-petrescu@umft.ro (M.B.-P.); iurciuc.stela@umft.ro (S.I.); 7 Department of Surgical Disciplines, Faculty of Medicine and Pharmacy, University of Oradea, 410073 Oradea, Romania; adrian.cote@didactic.uoradea.ro

**Keywords:** COVID-19, sepsis, IL-6, troponin, NT-proBNP, vaccination status, BMI, tocilizumab, mortality, prognostic biomarkers

## Abstract

(1) Background: COVID-19 sepsis, marked by hyperinflammation and cardiac injury, poses significant challenges in high-comorbidity populations. This prospective cohort study evaluates the prognostic value of IL-6, troponin, NT-proBNP, and radiological findings for mortality and unfavorable outcomes in a post-2022 Eastern European cohort. (2) Methods: At “Victor Babes” Hospital, Timisoara, Romania (September 2022–December 2024), 207 adults with COVID-19 sepsis (Sepsis-3 criteria) were enrolled. Baseline IL-6, troponin, NT-proBNP, CRP, PCT, D-dimers, and chest CT lung involvement were measured. Unfavorable outcomes (in-hospital death, ICU transfer, mechanical ventilation, or vasopressor use) were analyzed using logistic and linear regression. (3) Results: Among 207 patients (mean age: 68.7 years, 54.1% male), 52 (25.1%) experienced unfavorable outcomes. Multivariable analysis identified IL-6 (OR 1.016 per pg/mL, *p* = 0.013), troponin (OR 1.013 per ng/L, *p* = 0.017), NT-proBNP (OR 1.009 per pg/mL, *p* = 0.049), >50% lung involvement (OR 1.835, *p* = 0.011), unvaccinated status (OR 2.312, *p* = 0.002), and higher BMI (OR 1.112 per kg/m^2^, *p* = 0.005) as independent predictors of unfavorable outcomes. Tocilizumab use (n = 12) was associated with reduced mortality (*p* = 0.041). IL-6 (cut-off 39.0 pg/mL, AUC = 0.91) and troponin (cut-off = 111.3 ng/L, AUC = 0.88) showed strong predictive accuracy. (4) Conclusions: Elevated IL-6, troponin, NT-proBNP, severe lung involvement, unvaccinated status, and higher BMI predict adverse outcomes in COVID-19 sepsis. Tocilizumab may offer survival benefits, warranting larger trials. These findings support targeted risk stratification in high-comorbidity populations.

## 1. Introduction

Sepsis is a life-threatening condition resulting from a dysregulated host response to infection, responsible for approximately 11 million deaths annually worldwide [1,2]. In COVID-19, caused by SARS-CoV-2, sepsis manifests as a distinct viral form with severe hyperinflammation, endothelial damage, and thrombotic risk, especially in comorbid patients [3,4]. The pathophysiology involves cytokine imbalances, including TNF-α and IL-1β, leading to immune activation and tissue injury [5,6]. This cytokine storm drives acute respiratory distress and multi-organ failure in COVID-19 sepsis [7,8].

Interleukin-6 (IL-6), produced by monocytes, macrophages, and endothelial cells in response to infection, plays a key role in this inflammatory process. Triggered by signals like TNF-α, IL-6 drives acute-phase responses and organ damage through persistent inflammation [9,10,11]. High IL-6 levels correlate with disease severity, including risks of mechanical ventilation and mortality in COVID-19 [9,10]. Recent evidence confirms IL-6 as a predictor of severity and death in critically ill COVID-19 patients, suggesting its use for early risk assessment [11,12].

Other biomarkers, including C-reactive protein (CRP) and procalcitonin (PCT), indicate systemic inflammation and bacterial co-infection, often increasing with COVID-19 severity [13,14]. Elevated D-dimer levels signal coagulopathy and thrombosis, associated with poor outcomes in severe COVID-19 [15,16].

Cardiac involvement in COVID-19 sepsis, indicated by elevated troponin and N-terminal pro-B-type natriuretic peptide (NT-proBNP), is common and linked to myocardial injury [17,18]. Troponin increases due to viral invasion, hypoxia, or inflammatory damage, correlating with cardiovascular events and mortality [19,20]. NT-proBNP, reflecting cardiac stress, is elevated in severe COVID-19, even without prior heart failure, and predicts worse outcomes [12,14,20].

Severe COVID-19 also causes extensive lung damage, including diffuse alveolar injury and inflammation, often requiring oxygen or ventilation support [21,22]. Chest CT scans reveal ground-glass opacities, consolidation, and interstitial changes, aiding assessment of lung involvement [23,24].

Although prior studies have explored individual predictors of COVID-19 sepsis outcomes, few have integrated inflammatory, cardiac, and radiological markers with clinical factors such as vaccination status and body mass index in real-world, high-comorbidity settings. This study evaluates the prognostic roles of IL-6, troponin, NT-proBNP, chest CT lung involvement, vaccination status, and BMI in predicting mortality and unfavorable outcomes in a prospective Eastern European cohort with COVID-19 sepsis. By developing a composite multivariable model incorporating these factors, we aim to enhance risk stratification and inform clinical decisionmaking.

## 2. Materials and Methods

### 2.1. Study Design and Population

This prospective cohort study was conducted at the “Victor Babes” Infectious Diseases and Pneumology Hospital in Timisoara, Romania, from 1 September 2022to 31 December 2024. Adult patients (≥18 years) were prospectively enrolled in this study if (1) they had a confirmed COVID-19 diagnosis via a positive RT-PCR test; (2) they were diagnosed with sepsis at admission, per Sepsis-3 criteria [1], defined as a Sequential Organ Failure Assessment (SOFA) score ≥2 in the context of suspected or documented infection; and (3) the patient or their legal representative signed informed consent prior to study enrolment. Exclusion criteria were the following: (1) septic shock at admission (requiring mechanical ventilation or vasopressors); (2) incomplete data (e.g., missing imaging or biomarker data); and (3) refusal to sign informed consent for study participation (providing reason for refusal was not mandatory). Ethical approval was obtained from the Ethics Board of “Victor Babes” University of Medicine and Pharmacy, Timisoara (reference: 70/01.09.2022, revised: 2174/10.03.2023). This study was conducted in accordance with the Declaration of Helsinki. The study protocol was not prospectively registered in any database. No power analysis was conducted prior to patient enrolment.

### 2.2. Data Collection

At admission, patients underwent clinical evaluation, and the following data werecollected: age, sex, vaccination status, smoking status, alcohol consumption, body mass index (BMI), and comorbidities, and based on the comorbidity data, the Charlson Comorbidity Index (CCI) was calculated. Clinical data included symptom onset to admission duration, peripheric oxygen saturation (SpO2), and oxygen requirements. Oxygen supplementation was adjusted based on SpO2 targets (>92%) and clinical judgment, incorporating respiratory rate assessments; no standardized FiO2/SpO2 table was used, but escalation followed hospital protocols for progressive hypoxia. Chest computer tomography (CT) scans categorized lung involvement as>50%, 25–50%, and <25%. Laboratory biomarkers, including interleukin-6 (IL-6, pg/mL), troponin (ng/L), N-terminal pro-B-type natriuretic peptide (NT-proBNP, pg/mL), C-reactive protein (CRP, mg/L), procalcitonin (PCT, ng/mL), D-dimers (µg/mL), erythrocyte sedimentation rate (ESR), ferritin, and neutrophil-to-lymphocyte ratio (NLR), were collected at admission. Biomarker measurements were incomplete for 23 patients due to urgent clinical priorities (e.g., immediate resuscitation needs overriding routine lab draws; n = 15) or logistical issues (e.g., sample hemolysis or equipment downtime; n = 8), leading to their exclusion to ensure data completeness for IL-6, troponin, and NT-proBNP.

IL-6 was measured using the Quantikine HS ELISA kit (R&D Systems, Minneapolis, MN, USA), troponin via the Elecsys Troponin T hs assay (Roche Diagnostics, Basel, Switzerland), and NT-proBNP via the ElecsysproBNP II assay (Roche Diagnostics, Basel, Switzerland). CRP, PCT, and D-dimers were measured using automated immunoassays (turbidimetry for CRP, chemiluminescence for PCT, and latex-enhanced immunoturbidimetry for D-dimers). An electrocardiogram (EKG) assessed heart rate, ischemic changes, and hypertrophy.

Prospective data collection included treatment options during hospital stay, such as antivirals (e.g., remdesivir), antibiotics, corticosteroids, and tocilizumab. Remdesivir was administered as an antiviral agent in line with hospital protocols for moderate-to-severe COVID-19 cases. Antibiotics were used empirically for suspected bacterial co-infection. Corticosteroids were standard for inflammatory modulation. Tocilizumab, an IL-6 receptor antagonist, was administered to patients with severe COVID-19 sepsis, defined by IL-6 levels >40 pg/mL and oxygen requirements >15 L/min, based on clinical guidelines and physician discretion. The primary outcome was a composite adverse outcome, as follows: in-hospital death, ICU transfer, mechanical ventilation, or vasopressor use. Patients were stratified into favorable (no adverse outcome) and unfavorable (any adverse outcome) groups based on this composite criterion for subsequent analyses. Length of hospital stay (LOS) was defined as the duration of the acute hospitalization from admission to discharge or death, excluding any rehabilitation or chronic care periods.

### 2.3. Statistical Analysis

Descriptive statistics summarized characteristics. Continuous variables were assessed for normality using the Shapiro–Wilk test. Variables with normal distribution (e.g., oxygen saturation) were reported as means (SD), while non-normally distributed variables (e.g., age, biomarkers, days to admission, CCI, SOFA score, and BMI) were reported as medians (IQR) to ensure consistency in reporting. Categorical variables were presented as frequencies (%). Univariate comparisons employed t-tests for normally distributed data, Mann–Whitney U tests for non-normally distributed data, or chi-square/Fisher’s exact tests for categorical data. Multivariable logistic regression was performed on predictors significant in univariate analysis (*p* < 0.05). Model fit was assessed via the Hosmer–Lemeshow test. Odds ratios (ORs) were calculated per one-unit increase in continuous variables unless otherwise specified. For biomarkers, unit definitions were IL-6 in pg/mL, troponin in ng/L, NT-proBNP in pg/mL, and BMI in kg/m^2^. For categorical variables, reference groups were indicated in tables. Additional sensitivity analyses were performed, excluding tocilizumab-treated patients, to confirm robustness. Missing data were minimal (<5% for any variable) and handled by listwise deletion, as imputation was deemed unnecessary due to the low proportion of missing values. A composite risk model was developed by summing standardized scores of significant variables from the multivariable logistic regression (IL-6, troponin, NT-proBNP, >50% lung involvement, unvaccinated status, and BMI), and its performance was evaluated via ROC analysis. Receiver operating characteristic (ROC) curves were generated for key predictors (IL-6, troponin, NT-proBNP, BMI, and vaccination status) to estimate the area under the curve (AUC) and optimal cut-offs determined posthoc by Youden’s index for predicting unfavorable outcomes. These cut-offs (e.g., IL-6 39.0 pg/mL) were not predefined criteria for group inclusion but were used to assess predictive thresholds in exploratory analyses. Analyses used SPSS Statistics v26 (IBM Corp., Armonk, NY, USA), with *p* < 0.05 as significant.

## 3. Results

### 3.1. General Characteristics of Study Population

During the study period (1 September 2022–31 December 2024), 230 patients with COVID-19 sepsis were screened for eligibility. Of these, 207 met all inclusion criteria, including complete baseline measurements of IL-6, troponin, and NT-proBNP, and were included in the analysis. Twenty-three patients were excluded due to incomplete biomarker data. Baseline characteristics are summarized in Table 1.

The mean age was 69 (61–77) years, with 112 males (54.1%). Most patients (85.0%) were unvaccinated, while 31 (15.0%) were vaccinated. The mean time from symptom onset to admission was 4.2 days (SD 1.5). Smoking was reported in 64 patients (30.9%) and frequent alcohol consumption in 78 (37.7%). The mean body mass index (BMI) was 28.5 (26–31). The following comorbidities were prevalent: hypertension (81.6%), diabetes (34.8%), and coronary artery disease (20.3%). The mean Charlson Comorbidity Index (CCI) was 3 (2–4). Chest CT showed >50% lung involvement in 81 patients (39.1%), 25–50% in 74 (35.7%), and <25% in 52 (25.1%). The mean oxygen saturation (SpO2) was 90.1% (SD 5.7), with 95 patients (45.9%) requiring >15 L/min oxygen. The mean Sequential Organ Failure Assessment (SOFA score) was 6 (5–8). Baseline biomarkers indicated inflammation, as follows: interleukin-6 (IL-6) 30 (20–45), troponin 95 (65–130), N-terminal pro-B-type natriuretic peptide (NT-proBNP) 550 (400–750), C-reactive protein (CRP) 110 (80–140), procalcitonin (PCT) 2.0 (1.5–3.0), and D-dimers 1.0 (0.6–1.5). EKG abnormalities were observed in 55 patients (26.6%). Treatments included remdesivir (91.3%), antibiotics (85.5%), corticosteroids (98.6%), and tocilizumab (12 patients, 5.8%). Of the 28 patients meeting the criteria for tocilizumab (IL-6 > 40 pg/mL and oxygen > 15 L/min), 12 received it based on physician discretion; the remaining 16 did not due to contraindications or supply issues. Tocilizumab use was associated with reduced mortality (*p* = 0.041). Adverse outcomes occurred in 52 patients (25.1%), as follows: 42 ICU transfers, 38 required mechanical ventilation, and 28 died. Long-term functional outcomes (6 months) were not included among the study objectives and were not available for all patients.

### 3.2. Univariate Analysis of Factors Associated with Unfavorable Outcomes

**Figure 1:**
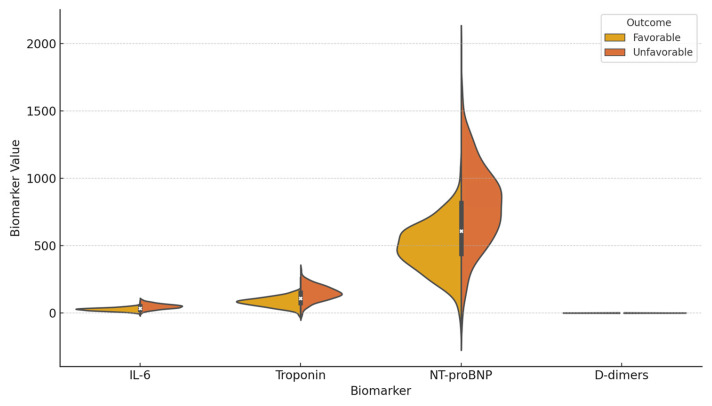
Violin plots comparing the distributions of IL-6 (pg/mL), troponin (ng/L), NT-proBNP (pg/mL), and D-dimers (µg/mL) between patients with favorable (n = 155) and unfavorable (n = 52) outcomes in COVID-19-related sepsis. The plots display medians, interquartile ranges, and density distributions. Significantly higher levels were observed in the unfavorable group for IL-6 (*p* = 0.012), troponin (*p* = 0.008), and NT-proBNP (*p* = 0.015), while no significant difference was found for D-dimers (*p* = 0.920).

The cohort (N = 207) was divided into the following two groups: favorable (n = 155) and unfavorable (n = 52). Table 2 compares the baseline characteristics of the favorable and unfavorable outcome groups. Significant differences were observed for age (higher in unfavorable, *p* = 0.0001), CCI (higher in unfavorable, *p* = 0.0002), CT severity (higher in unfavorable, *p* = 0.033), IL-6 (higher in unfavorable, *p* = 0.012), troponin (higher in unfavorable, *p* = 0.008), NT-proBNP (higher in unfavorable, *p* = 0.015), BMI (higher in unfavorable, *p* = 0.0003), and vaccination status (lower vaccinated in unfavorable, *p* = 0.001). No significant differences were found for diabetes (*p* = 0.452), frequent alcohol consumption (*p* = 0.681), sex (*p* = 0.763), smoking (*p* = 0.891), CRP (*p* = 0.950), PCT (*p* = 0.089), D-dimers (*p* = 0.920), EKG changes (*p* = 0.950), or oxygen flow >15 L/min (*p* = 0.072). SOFA score was higher in unfavorable (*p* = 0.025). Refer to Table 2 for numerical details. Figure 1 illustrates the distributions of key biomarkers among outcome groups using violin plots.

### 3.3. Multivariable Analysis of Predictors of Unfavorable Outcomes

**Figure 2:**
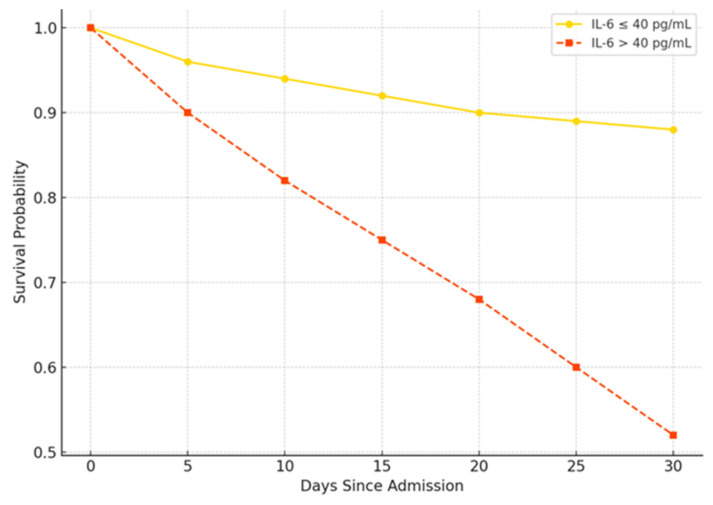
Kaplan–Meier survival curves stratified by IL-6 levels (high > 40 pg/mL vs. low ≤ 40 pg/mL), showing significant divergence (log-rank *p* < 0.01). The curve for high IL-6 demonstrates lower survival probability over time compared to low IL-6, with separation evident from early follow-up.

**Figure 3:**
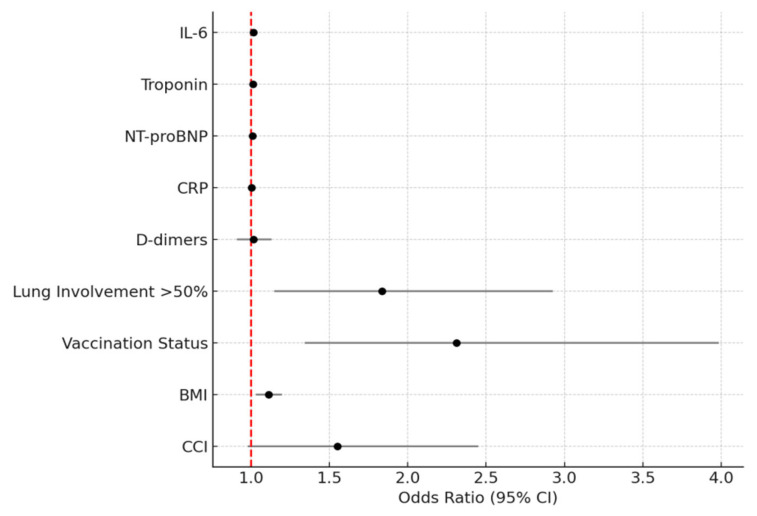
Forest plot of odds ratios from multivariable regression, visualizing the independent predictors of unfavorable outcomes with 95% confidence intervals (CIs). Horizontal lines represent CIs, black dots indicate OR point estimates, and the red dashed line marks the null effect (OR 1.0). Statistically significant predictors (e.g., IL-6, troponin, NT-proBNP, lung involvement >50%, vaccination status, and BMI) are located to the right of the null line.

Multivariable logistic regression identified independent predictors of unfavorable outcomes, as shown in Table 3. ORs for continuous variables are per unit increase (e.g., IL-6 per 1 pg/mL, troponin per 1 ng/L, NT-proBNP per 1 pg/mL, and BMI per 1 kg/m^2^). The Hosmer–Lemeshow test indicated a good fit (*p* = 0.065). Sensitivity analysis, excluding tocilizumab-treated patients, confirmed the results. Figure 2 displays the Kaplan–Meier survival curves stratified by IL-6 levels above or below 40 pg/mL, demonstrating significantly reduced survival in patients with elevated IL-6 (*p* = 0.001, log-rank test). Figure 3 presents the forest plot of the multivariate logistic regression model, illustrating the odds ratios and 95% CIs for each independent predictor of poor outcome.

To integrate predictors, a composite risk score was calculated by standardizing and summing significant variables (IL-6, troponin, NT-proBNP, lung involvement >50%, unvaccinated status, and BMI). The score predicted unfavorable outcomes (AUC = 0.93), outperforming individual markers. Combined elevated IL-6 (>39 pg/mL) and troponin (>111 ng/L) increased risk (OR 3.45, 95% CI 2.10–5.67, *p* < 0.001) compared to either alone. This composite model outperformed individual markers, supporting targeted risk stratification in clinical settings.

**Figure 4:**
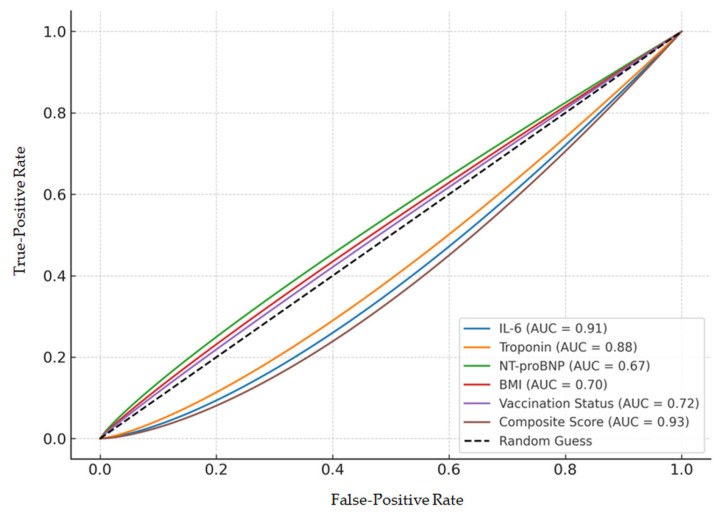
ROC curves for IL-6 (AUC = 0.91), troponin (AUC = 0.88), NT-proBNP (AUC = 0.67), BMI (AUC = 0.70), vaccination status (AUC = 0.72), and composite score (AUC = 0.93).

The predictive performance of individual biomarkers and clinical factors was evaluated using receiver operating characteristic (ROC) curves. IL-6 (AUC = 0.91), troponin (AUC = 0.88), NT-proBNP (AUC = 0.67), BMI (AUC = 0.70), and vaccination status (AUC = 0.72) all demonstrated varying degrees of prognostic accuracy. The discriminatory power of IL-6 for predicting unfavorable outcomes is demonstrated by ROC curves, as shown in Figure 4, with an AUC of 0.91 indicating strong predictive accuracy. A composite score integrating all significant predictors achieved the highest discriminative capacity (AUC = 0.93) for identifying patients at risk for unfavorable outcomes.

### 3.4. Linear Regression Analysis of Continuous Outcomes

Multiple linear regression analyses were conducted to assess predictors of length of hospital stay (LOS). Significant associations were observed for IL-6, troponin, >50% lung involvement, Charlson Comorbidity Index (CCI), unvaccinated status, and body mass index (BMI), independently of age. These findings are detailed in Table 4.

**Figure 5:**
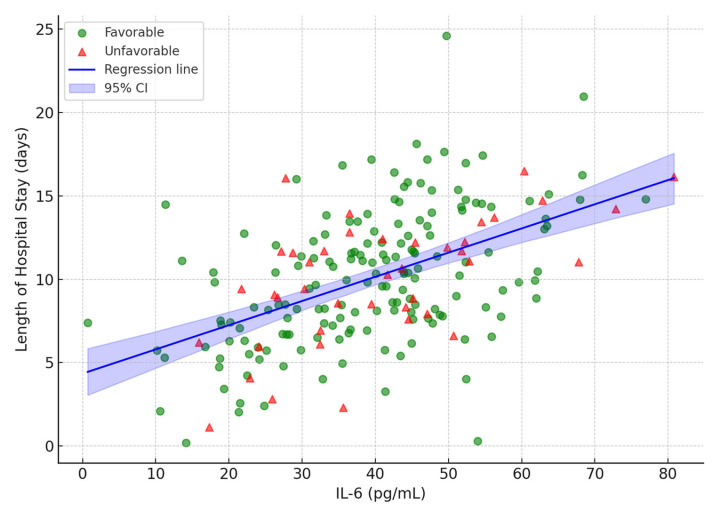
Scatterplot of IL-6 levels (pg/mL) versus length of hospital stay (days), with fitted regression line (solid blue) and 95% confidence bands (shaded). Points are stratified by outcome group (favorable: green circles; unfavorable: red triangles). The positive slope illustrates the association between elevated IL-6 and extended hospitalization (β = 0.123, *p* < 0.001).

Figure 5 presents the scatterplot of IL-6 versus LOS, demonstrating a positive association (β = 0.120, *p* < 0.001). See Supplementary Figures S1–S3 for similar scatterplots for troponin (β = 0.080, *p* < 0.001), NT-proBNP (β = 0.070, *p* = 0.002), and BMI (β = 0.300, *p* = 0.004), respectively. These results quantify how elevated values of each variable contribute to prolonged hospitalization and resource utilization.

A separate multiple linear regression for SOFA score showed comparable trends, with IL-6 (β = 0.044, *p* = 0.013), troponin (β = 0.027, *p* = 0.030), >50% lung involvement (β = 0.950, *p* = 0.010), CCI (β = 0.200, *p* = 0.012), unvaccinated status (β = −0.800, *p* = 0.003), and BMI (β = 0.110, *p* = 0.005) all independently associated with higher severity scores (adjusted R^2^ = 0.390).

The predictive performance of individual biomarkers and clinical factors was evaluated using receiver operating characteristic (ROC) curves. IL-6 (AUC = 0.91), troponin (AUC = 0.88), NT-proBNP (AUC = 0.67), BMI (AUC = 0.70), and vaccination status (AUC = 0.72) all demonstrated varying degrees of prognostic accuracy. A composite score integrating all significant predictors achieved the highest discriminative capacity (AUC = 0.93) for identifying patients at risk for unfavorable outcomes.

## 4. Discussion

In this prospective study of patients with COVID-19 sepsis, we assessed the prognostic value of IL-6, troponin, NT-proBNP, and clinical–radiological factors. The high-comorbidity cohort had prevalent hypertension (81.6%) and diabetes (34.8%). Key predictors included IL-6, troponin, NT-proBNP, >50% lung involvement, unvaccinated status, and elevated BMI, integrated into a composite model (AUC = 0.93) for improved risk stratification.

Elevated IL-6 independently predicted outcomes (OR 1.016 per pg/mL, *p* = 0.013) and longer LOS (β = 0.120, *p* < 0.001), with a 39.0 pg/mL cut-off (AUC = 0.91). Herold et al. (2020) reported elevated IL-6 levels predicting the need for mechanical ventilation (OR 2.85, 95% CI 1.8–3.7) [9]. Varga et al. (2025) confirmed IL-6’s role in sepsis mortality [11]. Tocilizumab (n = 12) reduced mortality (*p* = 0.041), consistent with Aziz et al. (2021) [25]. The discriminatory power of IL-6 for predicting unfavorable outcomes is demonstrated by ROC curves, as in Figure 5, with an AUC of 0.91 indicating strong predictive accuracy.

Troponin predicted outcomes (OR 1.013 per ng/L, *p* = 0.017) and LOS (β = 0.080, *p* < 0.001), with 111.3 ng/L cut-off (AUC = 0.88). 

Vrsalovic & Vrsalovic Presecki (2020) noted associations between cardiac troponins and increased risk of mechanical ventilation (OR 1.85, 95% CI 1.32-2.59) [20].Shi et al. (2020) linked cardiac injury to mortality (OR 3.75) [17]. Lombardi et al. (2020) emphasized the role of pre-existing comorbidities in amplifying troponin-associated mortality in COVID-19 patients [26]. Moreover, IL-6 levels predicted troponin elevation (β = 1.40 ng/L per pg/mL, *p* = 0.002, R^2^ = 0.21), indicating a potential mechanistic link between inflammation and cardiac injury in COVID-19-related sepsis.

NT-proBNP predicted outcomes (OR 1.009 per pg/mL, *p* = 0.049). Guo et al. (2020) reported elevated NT-proBNP levels associated with increased mortality risk (OR 5.12) [18]. Benhuri et al. (2022) noted threshold variability [27].

Unvaccinated status, assessed as a clinical risk factor, predicted outcomes (OR 2.312, *p* = 0.002) and longer LOS (β = −2.500, *p* = 0.002), with low vaccination (15.0%). Meslé et al. (2024) estimated vaccination prevented 1.6 million deaths [28]. Briciu et al. (2023) reported reduced mortality and severity in vaccinated Romanians [29]. Zhou et al. (2020) showed milder outcomes in vaccinated populations [4].

Higher BMI predicted outcomes (OR 1.112 per kg/m^2^, *p* = 0.005) and LOS (β = 0.300, *p* = 0.004). Kompaniyets et al. (2021) confirmed an increased mortality risk among obese patients with COVID-19, particularly in those under 65 years [30]. Poly et al. (2021) reported pooled OR 1.42 (95% CI 1.24–1.63) for mortality [31].

D-dimers did not predict outcomes (*p* = 0.930). Nemec et al. (2022) found no association [15]. Zhao et al. (2021) reported OR 3.45, possibly due to variant differences [16].

Regarding SARS-CoV-2 variants, this study did not perform variant typing, which is a limitation. Given the post-2022 enrollment period, the cohort likely included predominantly Omicron variants, which are associated with milder outcomes compared to earlier strains like Delta. However, persistent hyperinflammation in sepsis cases may explain the observed biomarker associations, consistent with variant-agnostic inflammatory pathways in severe disease. This lack of variant typing limits our ability to assess the impact of specific SARS-CoV-2 strains on outcomes, potentially affecting the generalizability of our findings.

Future studies should incorporate genomic sequencing to assess variant-specific impacts. Omicron’s lower virulence may attenuate some inflammatory responses, but hyperinflammation in sepsis likely persists across variants, explaining the robust biomarker associations observed.

Tocilizumab (n = 12) reduced mortality (*p* = 0.041) but was not analyzed as superior to other therapies like remdesivir (used in 91.3% as antiviral standard) or corticosteroids (98.6%). As an IL-6-targeted immunomodulator, tocilizumab was highlighted due to its alignment with elevated IL-6 findings and modern guidelines (e.g., WHO REACT meta-analysis, RR 0.83 for mortality reduction) [32]. It complements rather than replaces antivirals like remdesivir, per protocols for severe cases. Remdesivir and antibiotics were not analyzed as predictors due to their near-universal use (91.3% and 85.5%, respectively), which limited variability for statistical modeling, unlike tocilizumab, which was selectively administered based on IL-6 levels and clinical severity. The focus on tocilizumab was driven by its targeted mechanism against IL-6, a key predictor in our study, whereas remdesivir and corticosteroids were standard care, limiting their analysis as variables due to widespread use.

Tleyjeh et al. (2021) showed RR 0.83 with corticosteroids [33]. Derde et al. (2025) reported RR 0.89 [34]. Li et al. (2024) found no formulation difference [35]. Limitations include the small subgroup and potential selection bias.

Strengths include the prospective design and integrated model (Figure 2, Figure 3 and Figure 5; Table 3), explaining 48% of the LOS and 39% of the SOFA variances, supporting combined use for care allocation.

Limitations include the following: single center, small tocilizumab subgroup, no power analysis, low vaccination rate, no variant typing, and no IL-6 kinetics. Additionally, other potentially useful markers (e.g., lactate dehydrogenase and fibrinogen) were not evaluated in this study. Future studies should include multicenter validation, powered analyses, and long-term outcomes.

## 5. Conclusions

In patients with COVID-19 sepsis, elevated levels of IL-6, troponin, and NT-proBNP, along with severe lung involvement, unvaccinated status, and higher BMI, are independent predictors of adverse outcomes. A composite risk model integrating these factors improves prognostic accuracy. Preliminary findings from a small subgroup suggest potential survival benefits from tocilizumab therapy; however, due to the limited sample size and non-prospective selection, these results should be interpreted cautiously and warrant further investigation in larger, multicenter trials. Clinicians should prioritize early assessment of these biomarkers and clinical factors at admission to enable targeted risk stratification and optimize resource allocation for high-risk patients in resource-limited settings. Vaccination status should be routinely considered as a modifiable risk factor to inform preventive strategies.

## Figures and Tables

**Table 1:** Baseline characteristics of patients with COVID-19-associated sepsis.

Characteristic	Value (n = 207)
Demographics	
Age (years), Median (IQR)	69 (61–77)
Male Sex, n (%)	112 (54.1%)
Vaccination Status	
Unvaccinated, n (%)	176 (85.0%)
Vaccinated, n (%)	31 (15.0%)
Clinical Characteristics	
Days to Admission, Mean (SD)	4.2 (1.5)
Smoking, n (%)	64 (30.9%)
Frequent Alcohol Consumption, n (%)	78 (37.7%)
BMI, Median (IQR)	28.5 (26–31)
Comorbidities	
CCI Score, Median (IQR)	3 (2–4)
Hypertension, n (%)	169 (81.6%)
Diabetes, n (%)	72 (34.8%)
Coronary Artery Disease, n (%)	42 (20.3%)
CT Severity Score	
<25% Lung Involvement, n (%)	52 (25.1%)
25–50% Lung Involvement, n (%)	74 (35.7%)
>50% Lung Involvement, n (%)	81 (39.1%)
Oxygenation and Severity	
Oxygen Saturation at Baseline, Mean (SD)	90.1 (5.7)
Oxygen Flow >15 L/min, n (%)	95 (45.9%)
SOFA Score, Median (IQR)	6 (5–8)
Baseline Biomarkers, Median (IQR)	
IL-6 (pg/mL)	30 (20–45)
Troponin (ng/L)	95 (65–130)
NT-proBNP (pg/mL)	550 (400–750)
CRP (mg/L)	110 (80–140)
PCT (ng/mL)	2.0 (1.5–3.0)
D-dimers (µg/mL)	1.0 (0.6–1.5)
EKG Changes	
EKG Changes, n (%)	55 (26.6%)
Treatment, n (%)	
Remdesivir	189 (91.3%)
Antibiotics	177 (85.5%)
Corticosteroids	204 (98.6%)
Tocilizumab	12 (5.8%)
Length of Stay	
Length of Stay (days), Mean (SD)	14.3 (8.0)
Outcomes, n (%)	
ICU Admission	42 (20.3%)
Mechanical Ventilation	38 (18.4%)
Death	28 (13.5%)

**Table 2:** Baseline characteristics stratified by outcome.

Characteristic	Favorable (n = 155)	Unfavorable (n = 52)	*p*-Value
Demographics			
Age (years), Mean (SD); Median (IQR)	66.3 (10.4); 66 (59–74)	76.2 (9.8); 76 (69–83)	0.0001
Male Sex, n (%)	83 (53.5%)	29 (55.8%)	0.763
BMI, Mean (SD);Median (IQR)	27.3 (3.4); 27 (25–30)	30.5 (3.3); 30 (28–33)	0.0003
Vaccination Status, n (%)			0.001
Vaccinated	31 (20.0%)	0 (0.0%)	
Unvaccinated	124 (80.0%)	52 (100.0%)	
Comorbidities			
CCI, Mean (SD); Median (IQR)	2.8 (1.2); 3 (2–4)	3.8 (1.3); 4 (3–5)	0.0002
CT Severity, n (%)			
<25%	44 (28.4%)	8 (15.4%)	
25–50%	58 (37.4%)	16 (30.8%)	
>50%	53 (34.2%)	28 (53.8%)	
Oxygenation and Severity			
Oxygen Saturation, Mean (SD)	90.2 (5.9)	87.3 (8.1)	0.194
Oxygen Flow >15 L/min, n (%)	98 (63.2%)	43 (82.7%)	0.072
SOFA Score, Mean (SD); Median (IQR)	5.8 (2.1); 6 (4–7)	7.8 (2.5); 8 (6–10)	0.025
Baseline Biomarkers, Mean (SD), Median (IQR)			
IL-6 (pg/mL)	27.3(12.8);25(18–35)	48.7 (19.4); 45 (35–60)	0.012
Troponin (ng/L)	78.9(34.6);75(55–100)	145.2(56.3);140(110–180)	0.008
NT-proBNP (pg/mL)	489.3(189.7);480(350–600)	789.4 (298.2); 750 (600–950)	0.015
CRP (mg/L)	111.8(45.2);110(80–140)	111.8 (45.2); 110 (80–140)	0.950
PCT (ng/mL)	2.1 (1.1); 2.0 (1.4–2.8)	2.7 (1.4); 2.5 (1.8–3.5)	0.089
D-dimers (µg/mL)	1.1 (0.8); 1.0 (0.6–1.4)	1.1 (0.8); 1.0 (0.6–1.5)	0.920
EKG Changes			
EKG Changes, n (%)	41 (26.5%)	14 (26.9%)	0.950

**Table 3:** Multivariable logistic regression for predictors of unfavorable outcomes.

Variable	OR	95% CI	*p*-Value
CCI	1.550	0.980–2.452	0.060
IL-6	1.016	1.004–1.028	0.013
Troponin	1.013	1.003–1.023	0.017
NT-proBNP	1.009	1.000–1.018	0.049
CRP	1.002	0.992–1.012	0.910
D-dimers	1.015	0.910–1.132	0.930
Lung Involvement (>50%)	1.835	1.150–2.927	0.011
Vaccination Status (Unvaccinated vs. Vaccinated)	2.312	1.342–3.986	0.002
BMI	1.112	1.032–1.198	0.005

**Table 4:** Multiple linear regression for predictors of length of hospital stay.

Variable	β	95% CI	*p*-Value
IL-6 (per pg/mL)	0.120	0.078–0.162	<0.001
Troponin (per ng/L)	0.080	0.065–0.095	<0.001
D-dimers (per µg/mL)	0.150	−0.650–0.950	0.850
Lung Involvement (>50% vs. ≤50%)	2.650	1.290–4.010	<0.001
Age (per year)	0.045	−0.015–0.105	0.140
CCI	0.430	0.110–0.750	0.010
Vaccination Status (Unvaccinated vs. Vaccinated)	−2.500	−4.082–−0.918	0.002
BMI(per kg/m^2^)	0.300	0.100–0.500	0.004

## Data Availability

The original contributions presented in the study are included in the article, and further inquiries can be directed to the corresponding author.

## Supplementary Materials

The following supporting information can be downloaded at: https://www.mdpi.com/article/10.3390/v17091168/s1. Supplementary Figure S1. Scatterplot of troponin (ng/L) versus length of hospital stay (days), with fitted regression line and 95% confidence bands. Supplementary Figure S2. Scatterplot of NT-proBNP (pg/mL) versus length of hospital stay (days), with fitted regression line and 95% confidence bands. Supplementary Figure S3. Scatterplot of BMI (kg/m^2^) versus length of hospital stay (days), with fitted regression line and 95% confidence bands.

## Abbreviations

The following abbreviations are used in this manuscript:

NT-proBNP	N-Terminal Pro-B-Type Natriuretic Peptide
IL-6	Interleukin-6
CRP	C-Reactive Protein
PCT	Procalcitonin
SpO2	Oxygen Saturation Measured by Pulse Oximetry
CT	Computed Tomography
ICU	Intensive Care Unit
LOS	Length of Stay
CCI	Charlson Comorbidity Index
BMI	Body Mass Index
AUC	Area Under the Curve (ROC)
ROC	Receiver Operating Characteristic curve
COVID-19	Coronavirus Disease 2019
SARS-CoV-2	Severe Acute Respiratory Syndrome Coronavirus 2
MV	Mechanical Ventilation
SOFA	Sequential Organ Failure Assessment
AUROC	Area Under the Receiver Operating Characteristic Curve
OR	Odds Ratio
CI	Confidence Interval
SD	Standard Deviation
IQR	Interquartile Range
*p*	*p*-Value
