# Supplementary material for: Predicting COVID-19 Sepsis Outcomes: Roles of IL-6, Cardiac Biomarkers, Clinical Factors, and Vaccination Status and Exploratory Analysis of Tocilizumab Therapy in an Eastern European Cohort
Source: Viruses. 2025 Aug 27;17(9):1168. doi: 10.3390/v17091168 (PMC12474509; doi:10.3390/v17091168)
Supplement: Supplementary file 1 [file viruses-17-01168-s001.zip › Supplementary Materials.pdf]

Supplementary Materials

Supplementary Figure S1. Scatterplot of troponin (ng/L) versus length of hospital stay (days), with fitted regression line and 95% confidence bands.
Supplementary Figure S2. Scatterplot of NT-proBNP (pg/mL) versus length of hospital stay (days), with fitted regression line and 95% confidence bands.
Supplementary Figure S3. Scatterplot of BMI (kg/m²) versus length of hospital stay (days), with fitted regression line and 95% confidence bands.

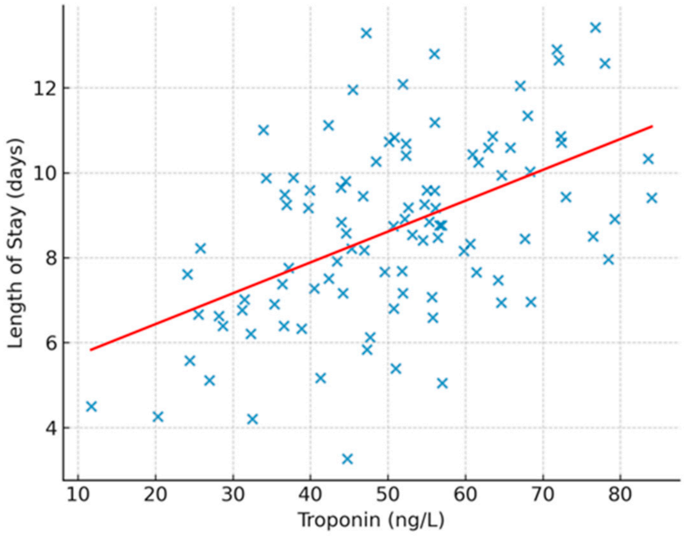

Supplementary Figure S1. Scatterplot of troponin (ng/L) versus length of hospital stay (days), with fitted regression line and 95% confidence bands.

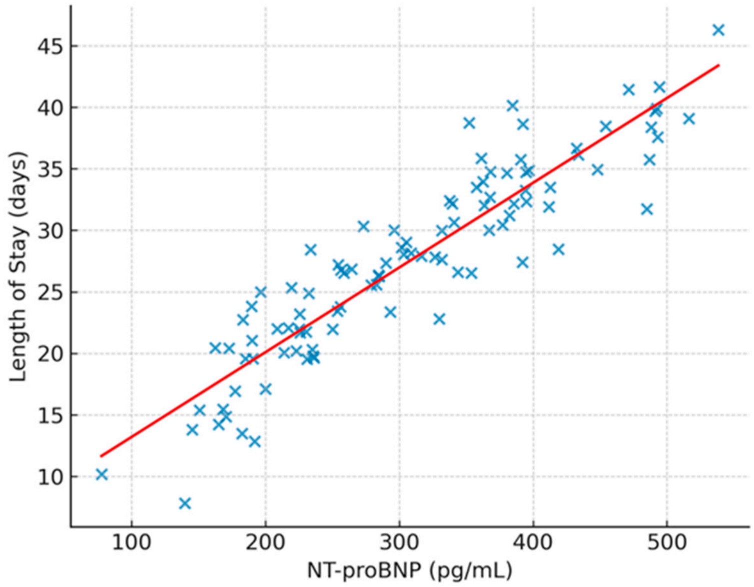

Supplementary Figure S2. Scatterplot of NT-proBNP (pg/mL) versus length of hospital stay (days), with fitted regression line and 95% confidence bands.

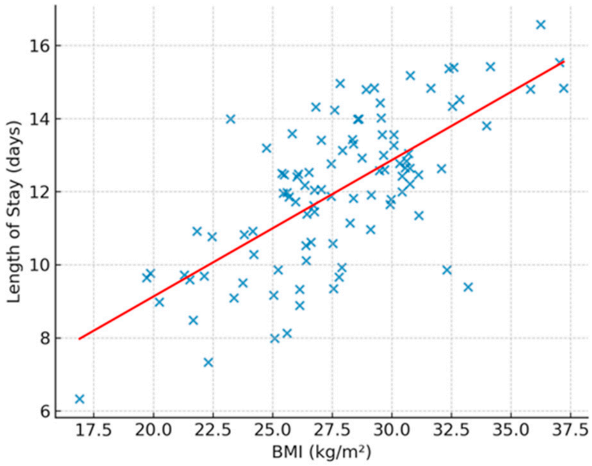

Supplementary Figure S3. Scatterplot of BMI (kg/m²) versus length of hospital stay (days), with fitted regression line and 95% confidence bands.
